# Supplementary material for: Dynamic modulation of activity in cerebellar nuclei neurons during pavlovian eyeblink conditioning in mice
Source: eLife. 2017 Dec 15;6:e28132. doi: 10.7554/eLife.28132 (PMC5760204; doi:10.7554/eLife.28132)
Supplement: Figure 5—source data 1. [file elife-28132-fig5-data1.docx]

| **Fig. 5 Source Data.** DCN model properties* | |
| --- | --- |
| # PCs per CN cell | 30 |
| φ, firing threshold | -38.8 mV |
| C_m_, membrane capacitance | 122.3 pF |
| g_leak_, max leak conductance | 1.63 nS |
| E_leak_, leak reversal potential | -56 mV |
| g_AMPA_, max AMPA conductance | 50 nS |
| g_NMDA_, max NMDA conductance | 25.8 nS |
| g_ex_base_, baseline excitatory conductance | 12 nS |
| E_ex_, excitatory reversal potential | 0 mV |
| g_inh_, max inhibitory conductance | 5 nS |
| E_inh_, inhibitory reversal potential | -70 mV |
| g_ahp_, max after-hyperpolarization | 50 nS |
| E_ahp_, after-hyperpolarization reversal potential- | -70 mV |
| τ_AMPA_, AMPA time constant | 9.9 ms |
| τ_NMDA_, NMDA time constant | 30.6 ms |
| τ_inh_, inhibitory time constant | 2.4 ms |
| τ_ahp_, after-hyperpolarization time constant | 2.5 ms |
| *Sources: Yamazaki and Tanaka, 2007; Person and Raman, 2012 | |
