# Supplementary material for: Dynamic modulation of activity in cerebellar nuclei neurons during pavlovian eyeblink conditioning in mice
Source: eLife. 2017 Dec 15;6:e28132. doi: 10.7554/eLife.28132 (PMC5760204; doi:10.7554/eLife.28132)
Supplement: Supplementary file 1. [file elife-28132-supp1.docx]

| **Supplementary File 1.** Linear (mixed) model summaries. | | | |
| --- | --- | --- | --- |
|  | | | |
| **Fig. 2B:** For outcome variable **%** **Eyelid closure at US onset** with random intercepts and slopes per **IpN cell** (showing significant spike-eyelid correlations, n = 17, original dataset): | | | |
| **Fixed effect** | **Coefficient** | **F-test** | **P** |
| Intercept | 6.9 ± 2.1 | F_1,556_ = 10.7 | 0.0011 |
| Spike facilitation (Hz) | 0.49 ± 0.07 | F_1,1556_ = 44.97 | <0.0001 |
|  | | | |
| **Fig. 2F:** For outcome variable **%** **Eyelid closure at US onset** with random intercepts and slopes per **IpN cell** (showing significant spike-eyelid correlations, n = 49, second dataset): | | | |
| **Fixed effect** | **Coefficient** | **F-test** | **P** |
| Intercept | 7.57 ± 2.47 | F_1,2071_ = 9.36 | 0.0022 |
| Spike facilitation (Hz) | 0.64 ± 0.04 | F_1,2071_ = 265.12 | <0.0001 |
|  | | | |
| **Fig. 2J:** For outcome variable **%** **Eyelid closure at US onset** with random intercepts and slopes per **IpN cell** (showing significant spike-eyelid correlations, n = 3): | | | |
| **Fixed effect** | **Coefficient** | **F-test** | **P** |
| Intercept | 10.5 ± 5.1 | F_1,182_ = 4.29 | 0.0397 |
| Spike suppression (Hz) | -0.27 ± 0.12 | F_1,182_ = 4.96 | 0.0272 |
|  | | | |
| **Fig. 2N:** For outcome variable **%** **Eyelid closure at US onset** with random intercepts per **IpN cell** (showing significant spike-eyelid correlations, n = 8): | | | |
| **Fixed effect** | **Coefficient** | **F-test** | **P** |
| Intercept | 32.3 ± 3.5 | F_1,306_ = 83.12 | <0.0001 |
| Spike suppression (Hz) | -1.01 ± 0.1 | F_1,306_ = 104.19 | <0.0001 |
|  | | | |
| **Fig. 6D.** For outcome variable **Post-US peak (Hz)** with random intercepts and slopes per **IpN cell** (showing significant correlations between post-US peak and CR amplitude; n = 15): | | | |
| **Fixed effect** | **Coefficient** | **F-test** | **P** |
| Intercept | 192.5 ± 14.3 | F_1,559_ = 183.6 | <0.0001 |
| % Eyelid closure at US onset | -1.79 ± 0.36 | F_1,559_ = 24.9 | <0.0001 |
|  | | | |
| **Fig. 6H.** For outcome variable **US peak (Hz)** across **IpN cells** (showing significant correlations between post-US trough and CR amplitude; n = 4): | | | |
| **Fixed effect** | **Coefficient** | **T-test** | **P** |
| Intercept | 8.88 ± 1.86 | T_184_ = 4.76 | <0.0001 |
| % Eyelid closure at US onset | 0.66 ± 0.06 | T_184_ = 11.03 | <0.0001 |
|  | | | |
| **Fig. 7B.** For outcome variable **2^nd^ US peak (Hz)** with random intercepts and slopes per **IpN cell** (showing significant correlations between post-US peak and secondary post-US peak, n = 14): | | | |
| **Fixed effect** | **Coefficient** | **F-test** | **P** |
| Intercept | 76.2 ± 6.96 | F_1,549_ = 119.9 | <0.0001 |
| Post-US peak (Hz) | 0.31 ± 0.04 | F_1,549_ = 65.6 | <0.0001 |
