# Supplementary material for: Dynamic modulation of activity in cerebellar nuclei neurons during pavlovian eyeblink conditioning in mice
Source: eLife. 2017 Dec 15;6:e28132. doi: 10.7554/eLife.28132 (PMC5760204; doi:10.7554/eLife.28132)
Supplement: Transparent reporting form [file elife-28132-transrepform.docx]

***eLife’s* transparent reporting form**

We encourage authors to provide detailed information *within their submission* to facilitate the interpretation and replication of experiments. If you have any questions, please contact us: [editorial@elifesciences.org](mailto:editorial@elifesciences.org).

**Sample-size estimation**

- You should state whether an appropriate sample size was computed when the study was being designed
- You should state the statistical method of sample size computation and any required assumptions
- If no explicit power analysis was used, you should describe how you decided what sample (replicate) size (number) to use

Please outline where this information can be found within the submission (e.g., page numbers or figure legends), or explain why this information doesn’t apply to your submission:

Previously reported effect sizes from Ten Brinke et al. (2015) helped evaluate the appropriateness of the sample sizes and are mentioned on p. 24. Other than that, sample sizes were mostly defined by ethical and biological constraints, rather than by expected effect sizes for the broad range of neuronal responses and correlations among themselves and with behavior.

**Replicates**

- You should report how often each experiment was performed
- You should include a definition of biological versus technical replication
- The data obtained should be provided and sufficient information should be provided to indicate the number of independent biological and/or technical replicates
- If you encountered any outliers, you should describe how these were handled
- Criteria for exclusion/inclusion of data should be clearly stated
- High-throughput sequence data should be uploaded before submission, with a private link for reviewers provided (these are available from both GEO and ArrayExpress)

Please outline where this information can be found within the submission (e.g., page numbers or figure legends), or explain why this information doesn’t apply to your submission:

The numbers of recordings, and their inclusion criteria, were detailed In the Results on p. 6 and Figure 1; number of subjects in the Methods on p. 22. The approach to outliers is detailed on p. 26-27. Technical replication was not feasible in our experiments, as every trial was only recorded once, and each trial represented a different neuronal context.

**Statistical reporting**

- Statistical analysis methods should be described and justified
- Raw data should be presented in figures whenever informative to do so (typically when N per group is less than 10)
- For each experiment, you should identify the statistical tests used, exact values of N, definitions of center, methods of multiple test correction, and dispersion and precision measures (e.g., mean, median, SD, SEM, confidence intervals; and, for the major substantive results, a measure of effect size (e.g., Pearson's r, Cohen's d)
- Report exact p-values wherever possible alongside the summary statistics and 95% confidence intervals. These should be reported for all key questions and not only when the p-value is less than 0.05.

Statistical tests are described and justified on p. 26-27, and in Table. Raw data is included as much as possible across all figures, and the identity and necessary measures of statistical tests are given directly in the text of the Results section (p. 6-14). P-values are presented exactly for all findings, with one exception in the case of a non-key side observation on p. 10.

Please outline where this information can be found within the submission (e.g., page numbers or figure legends), or explain why this information doesn’t apply to your submission:

(For large datasets, or papers with a very large number of statistical tests, you may upload a single table file with tests, Ns, etc., with reference to page numbers in the manuscript.)

**Additional data files (“source data”)**

- We encourage you to upload relevant additional data files, such as numerical data that are represented as a graph in a figure, or as a summary table
- Where provided, these should be in the most useful format, and they can be uploaded as “Source data” files linked to a main figure or table
- Include model definition files including the full list of parameters used
- Include code used for data analysis (e.g., R, MatLab)
- Avoid stating that data files are “available upon request”

Please indicate the figures or tables for which source data files have been provided:

Two main Matlab variables are included: “Cells” is a table containing for all recordings the average of the parameters used in the study. “Trials” is a structure containing for each parameter the name and a cell array with all valid trial values per cell. Most of the correlational analyses described in the text can be recreated using these variables, as well as those visualized in Fig. 1N, O; Fig. 2C, D; Fig. 6C, D; Fig. 7D, E, H, I; and Fig. 8C, D. Model parameters for the data in Fig. 5 are included in Table.
